# Comparison of Postoperative Results With Prognostic Nutritional Index for Lumbar Disc Herniation

**DOI:** 10.7759/cureus.60584

**Published:** 2024-05-19

**Authors:** Hayato Kinoshita, Michio Hongo, Eiji Abe, Takashi Kobayashi, Yuji Kasukawa, Kazuma Kikuchi, Daisuke Kudo, Ryota Kimura, Yuichi Ono, Naohisa Miyakoshi

**Affiliations:** 1 Orthopedic Surgery, Honjo Daiichi Hospital, Yurihonjo, JPN; 2 Physical Therapy, Akita University Graduate School of Medicine, Akita, JPN; 3 Orthopedic Surgery, Johto Orthopedic Clinic, Akita, JPN; 4 Orthopedic Surgery, Akita Kousei Medical Center, Akita, JPN; 5 Orthopedic Surgery, Akita University Graduate School of Medicine, Akita, JPN; 6 Orthopedic Surgery, Yuri Kumiai General Hospital, Yurihonjo, JPN

**Keywords:** lumbar disc herniation, postoperative complications, total peripheral lymphocyte count, serum albumin, prognostic nutritional index (pni)

## Abstract

Introduction: The prognostic nutritional index (PNI) is an immune-nutritional index simply provided by a blood test. We retrospectively compared the postoperative outcomes of patients with lumbar disc herniation divided into two groups according to the PNI.

Materials and methods: Seventy-three patients who underwent surgery at our hospital were included in the study. All patients had herniation between one of the L3/4, L4/5, or L5/S intervertebral discs and underwent one posterior lumbar interbody fusion. These patients were divided into two groups: patients with a PNI of <50 (poorly nourished (PN) group) and patients with a PNI of ≥50 (well-nourished (WN) group). Evaluation items included patient background characteristics, operative time, blood loss, postoperative complications, and length of hospital stay.

Results: The results showed that the body mass index was significantly higher in the WN group than in the PN group (p=0.0221). The rates of collagen disease, steroid use, and postoperative complications were significantly higher (p=0.0475, p=0.0073, and p=0.0211, respectively) and the length of hospital stay was significantly longer (p=0.021) in the PN group than in the WN group.

Conclusion: In conclusion, this study indicates that postoperative complications and the length of hospital stay are significantly worse in PN patients than in WN patients.

## Introduction

The prognostic nutritional index (PNI) is an immune-nutritional index simply provided by a blood test. Onodera et al. used the formula PNI = serum albumin level (g/dL) × 10 + 0.005 × total peripheral lymphocyte count (/µL) in patients with gastrointestinal cancer [1]. In the field of orthopedics, Hanada et al. recently reported the relationship between PNI and postoperative aseptic wound complications in patients who underwent total knee arthroplasty [2].

Oe et al. divided their cohort of 285 adult patients with spinal deformity into two groups according to their PNI and concluded that the postoperative complications of the patients with a PNI of <50 were significantly higher than those of patients with a PNI of ≥50 [3]. Based on this report, Kurosu et al. examined the preoperative PNI in patients undergoing thoracolumbar spine surgery [4]. They divided their patients into two groups for comparison: those with a PNI of ≥50 and those with a PNI of <50. Their results showed a correlation between the PNI and postoperative outcomes. Furthermore, they cited variation in surgical invasiveness and comorbidity bias as limiting factors.

Based on the above-mentioned reports, we investigated whether preoperative PNI is associated with postoperative outcomes by comparing patients with as homogeneous as possible backgrounds and surgical invasiveness.

## Materials and methods

We enrolled 73 patients who underwent surgery at our hospital from January 2017 to January 2021. All patients had herniation between one of the L3/4, L4/5, or L5/S intervertebral discs. We performed a single posterior lumbar interbody fusion (PLIF) on each lesion. We performed PLIF for lumbar disc herniation when one of the following three conditions was met. (i) There was a huge herniation at the median aspect of the spinal canal and sagittalization of the facet joints, which would have caused injury to the dura mater or nerve when the dura mater was retracted as well as instability of the lumbar spine because of bone resection near the facet joint, even if the hernia were removed by fenestration only. (ii) The patient was undergoing reoperation, and a long period of time had elapsed since the initial operation. The usual approach, such as endoscopic or open discectomy, is considered to have a high risk of dural or nerve damage because of adhesions between the dura mater and surrounding tissue. (iii) Herniation with local kyphosis of the lumbar spine was present because of disc degeneration, and correction of spinal alignment was performed simultaneously with herniotomy.

The patients were divided into two groups: patients with a PNI of <50 (n=29) constituted the poorly nourished (PN) group, and patients with a PNI of ≥50 (n=43) constituted the well-nourished (WN) group. Evaluation items included patient background characteristics (age, sex, body mass index (BMI), comorbidities, liver and kidney function, steroid use, and details of hernia), types of pedicle screws, operative time, blood loss, postoperative complications, and length of hospital stay. All postoperative complications except surgical site infection were evaluated within one month after surgery. Surgical site infection was evaluated until one year after surgery.

Data regarding the ratios of sex, details of hernias, comorbidities, steroid use, types of pedicle screws, and postoperative complications were compared using the chi-squared test. Age at surgery, BMI, liver and kidney function, operative time, blood loss, and length of hospital stay were compared using an independent t-test after checking for equal variances. Values of p<0.05 were considered statistically significant.

Multiple regression analysis was performed with postoperative complications or hospital stay as the dependent variable and age, BMI, operative time, blood loss, postoperative complications, PNI, and length of hospital stay or postoperative complications as independent variables.

All statistical analyses were performed using Statistical Package for the Biosciences software (Nankodo, Tokyo, Japan) [5].

This was a case-control study. We obtained informed consent from all patients in accordance with the Declaration of Helsinki. This study was approved by the Ethics Committee of Akita Kousei Medical Center (approval code: 188).

## Results

Regarding patient background characteristics, the WN group had a higher BMI and a lower percentage of collagen disease and prednisolone use than the PN group (p=0.0221, p=0.0475, and p=0.0073, respectively) (Table 1).

**Table 1:** Patients’ background characteristics Data are presented as mean ± standard deviation or n. *Unpaired Student’s t-test #Chi-squared test WN, well-nourished; PN, poorly nourished; PNI, prognostic nutritional index; BMI, body mass index; AST, aspartate transaminase; ALT, alanine transaminase; BUN, blood urea nitrogen; Cre, creatinine

	WN group (n=43)	PN group (n=29)	P value
PNI	54.7±3.7	46.5±3.8	<0.0001*
Age	67.0±8.2	68.0±13.4	0.718
Sex (Male/Female)	25/18	17/12	0.839
BMI	25.7±4.3	23.6±3.2	0.022*
Hypertension	21	18	0.388
Diabetes	12	6	0.677
Cerebrovascular disease	3	2	0.942
Heart disease	4	2	0.942
Respiratory disease	2	4	0.264
Liver disease	3	2	0.646
Renal disease	0	3	0.12
Malignant tumor	2	3	0.646
Psychoneurotic disease	2	2	0.907
Autoimmune disorder	0	4	0.048^#^
Steroid oral medication	0	6	0.007^#^
AST (IU/L)	26.9±15.3	23.1±8.7	0.188
ALT (IU/L)	28.6±18.2	21.8±12.8	0.085
BUN (mg/dL)	16.6±5.3	17.7±7.1	0.471
Cre (mg/dL)	0.77±0.17	1.05±1.30	0.262

Other background data, including details of hernias, were adjusted without significant differences. Surgical invasion according to types of pedicle screws, operative time, and blood loss also showed no significant differences between the groups (Table 2).

**Table 2:** Details of hernia and operation Data are presented as n, n (%), or mean ± standard deviation. WN, well-nourished; PN, poorly nourished; PS, pedicle screw; PPS, percutaneous pedicle screw

	WN group (n=43)	PN group (n=29)	P value
Level of herniation (L3/4:L4/5:L5/S)	7:30:6	7:18:4	0.703
Reoperation Cases (%)	10/43 (23.3%)	7/29 (24.1%)	0.844
Approach (PS:PPS)	32:11	23:6	0.844
Operation time (min)	136±24	135±22	0.796
Blood loss (g)	103±63	91±57	0.420

Based on these background data, postoperative complications and the length of hospital stay were higher in the PN group than in the WN group (p=0.008 and p=0.021, respectively) (Table 3).

**Table 3:** Complications and length of hospital stay Data are presented as n or mean ± standard deviation. *Chi-squared test **Unpaired Student’s t-test WN, well-nourished; PN, poorly nourished; SSI, surgical site infection

	WN group (n=43)	PN group (n=29)	P value
Delirium	1	4	0.152
Pneumonia	0	1	0.842
Urinary tract infection	1	0	0.842
Diarrhea	1	3	0.351
Ischemic colitis	0	1	0.842
SSI	0	1	0.842
Total complications	3	10	*0.008
Hospital stay	22±13	29±15	**0.021

The multiple regression analysis showed no significant correlations using postoperative complications and hospital stay as the dependent variables. However, in the multiple regression analysis using the PNI as the dependent variable and age, BMI, operative time, blood loss, postoperative complications, and length of hospital stay as independent variables, the length of hospital stay was negatively correlated with the PNI (standard regression coefficient=−0.31, t value=−2.63, p=0.011). No clear correlation was found for other factors.

## Discussion

Preoperative assessment of nutrition is considered one of the important prognostic indicators for various surgical procedures. Various nutritional risk indicators have been reported in the past, including the geriatric nutritional risk index (GNRI), PNI, and controlling nutritional status (CONUT) score [1,6,7].

Cong and Chunwei compared these three nutritional scores in predicting postoperative complications after pancreaticoduodenectomy [8]. They concluded that the PNI predicted the occurrence and severity of postoperative complications better than the GNRI and CONUT score in their patient cohort. The present study showed that the PNI was useful in predicting the outcome of postoperative complications and the length of hospital stay in patients who underwent surgery for lumbar disc herniation.

Li et al. compared postoperative outcomes of degenerative disease-correcting and deformity-correcting spinal surgeries using the serum albumin concentration alone [9]. The authors concluded that hypoalbuminemia was a significant risk factor for complications after spinal surgeries. Further research is needed to verify which nutritional index gives a more accurate prognosis in patients undergoing spinal surgeries.

In the present study, there were more patients with autoimmune disorders in the PN group than in the WN group. Ahn et al. reported that the disease activity of systemic lupus erythematosus (SLE) was associated with PNI [10]. The PNI of patients with active SLE was lower than that of patients with inactive SLE.

One issue of concern is the number to set as the boundary of the PNI. Onodera et al. used the PNI to analyze 189 patients undergoing gastrointestinal surgery [1]. They suggested that resection and anastomosis of the gastrointestinal tract can be safely practiced when the PNI is >45. If the PNI is <40, this surgical approach may be contraindicated. Cong and Chunwei divided patients with pancreatic cancer and periampullary neoplasm into three nutritional groups: no nutritional risk (PNI>38), moderate nutritional risk (35≤PNI≤38), and severe nutritional risk (PNI<35). All of their patients were surgical patients with gastrointestinal diseases, whose nutritional status tends to be lower overall than that of patients with orthopedic diseases [8]. Regarding orthopedic disease, Hanada et al. suggested that the PNI of patients undergoing total knee arthroplasty who developed a postoperative aseptic wound problem was significantly lower than that of patients who did not [2]. Unlike reports on gastrointestinal diseases, the PNI in both groups was approximately 50 (51.0±4.6 vs. 48.2±5.7). Some studies have shown significant differences in complications after spinal surgery when patients are grouped by a PNI of 50 [3,4]. However, even in terms of orthopedic disorders, some patients are expected to be undernourished, such as those with osteosarcoma or metastatic bone tumors or those who are candidates for limb amputation. It is therefore necessary to group the PNI according to each individual case. In the present study, it was useful to divide patients with lumbar herniation into two groups according to a PNI of <50 and ≥50 to predict postoperative complications and the length of hospital stay.

If the PNI can help predict postoperative complications and the length of hospital stay, our next question of interest would be whether preoperative nutritional correction can reduce postoperative harm. Oe et al. reported that preoperative nutritional intervention would reduce postoperative complications in malnourished patients with adult spinal deformity [11]. Further research is needed to determine whether preoperative nutritional correction can reduce postoperative complications and hospital stay in patients with lumbar disc herniation.

This study has a limitation. The limitation is the small number of patients. Further investigations are needed to increase the number of cases in the future.

## Conclusions

Patients with lumbar disc herniations who underwent PLIF were studied using the PNI as a criterion. Despite the fact that patient background and surgical technique were largely uniform in this study, patients with a PNI of ≤50 had significantly more postoperative complications and longer hospital stays than those with a PNI of >50. In these patients, PNI may be useful in predicting postoperative outcomes.
